# A Systematic Review of Fetal Genes as Biomarkers of Cardiac Hypertrophy in Rodent Models of Diabetes

**DOI:** 10.1371/journal.pone.0092903

**Published:** 2014-03-24

**Authors:** Emily J. Cox, Susan A. Marsh

**Affiliations:** 1 Graduate Program in Pharmaceutical Sciences, College of Pharmacy, Washington State University, Spokane, Washington, United States of America; 2 Department of Experimental and Systems Pharmacology, College of Pharmacy, Washington State University, Spokane, Washington, United States of America; Loyola University Chicago, United States of America

## Abstract

Pathological cardiac hypertrophy activates a suite of genes called the fetal gene program (FGP). Pathological hypertrophy occurs in diabetic cardiomyopathy (DCM); therefore, the FGP is widely used as a biomarker of DCM in animal studies. However, it is unknown whether the FGP is a consistent marker of hypertrophy in rodent models of diabetes. Therefore, we analyzed this relationship in 94 systematically selected studies. Results showed that diabetes induced with cytotoxic glucose analogs such as streptozotocin was associated with decreased cardiac weight, but genetic or diet-induced models of diabetes were significantly more likely to show cardiac hypertrophy (P<0.05). Animal strain, sex, age, and duration of diabetes did not moderate this effect. There were no correlations between the heart weight:body weight index and mRNA or protein levels of the fetal genes α-myosin heavy chain (α-MHC) or β-MHC, sarco/endoplasmic reticulum Ca^2+^-ATPase, atrial natriuretic peptide (ANP), or brain natriuretic peptide. The only correlates of non-indexed heart weight were the protein levels of α-MHC (Spearman's ρ = 1, P<0.05) and ANP (ρ = −0.73, P<0.05). These results indicate that most commonly measured genes in the FGP are confounded by diabetogenic methods, and are not associated with cardiac hypertrophy in rodent models of diabetes.

## Introduction

Activation of the fetal gene program (FGP) in the adult heart occurs after cardiac insults and is ubiquitously used as a biomarker of cardiac hypertrophy [1], [2]. Diabetic cardiomyopathy is partly characterized by ventricular hypertrophy [3], [4]; therefore, the FGP is commonly used as an indicator of diabetic cardiomyopathy in rodent models of diabetes. However, many studies show that fetal genes are unchanged or downregulated in these animal models. For example, rodent models of diabetes show lower circulating serum levels [5]–[7], lower protein levels in cardiac tissue [8], [9], and lower cardiac transcript levels [8]–[16] of two commonly measured fetal genes, atrial and brain natriuretic peptide (ANP and BNP). We have also shown previously that the presence of type 2 diabetes in mice blocks cardiac expression of ANP in response to hypertrophic stimuli [17]. Another measure of FGP activation in the adult heart is a decrease in the expression of α- relative to β-myosin heavy chain (MHC); however, it has been reported that this ratio is actually increased in type 2 diabetic *db/db* mouse hearts, and that this increase depends on the duration of diabetes [12].

To our knowledge it has not been shown that fetal genes are consistent markers of cardiac hypertrophy in rodent models of diabetes. Therefore, this systematic review was performed to determine whether these animals show higher expression of fetal genes in the heart, and whether these genes correlate with cardiac weight. The results of this analysis show that most fetal genes do not correlate with cardiac hypertrophy in diabetic animals, and that methods of diabetogenicity significantly moderate the development of cardiac hypertrophy and the expression of fetal genes.

### Overview of fetal genes

The following genes are some of the most commonly measured members of the FGP that are often used as indicators of cardiac hypertrophy.

#### Serca2

The sarcoplasmic reticulum Ca^2+^ ATPase 2 (Serca2) is responsible for re-uptake of calcium into the SR following contraction of the sarcomere, thus permitting muscle relaxation. Levels of Serca2 increase throughout fetal development of the mammalian myocardium and are maintained in adulthood [18]. A decrease in Serca2 expression is observed in the diabetic heart [19]–[21] and may underlie diastolic dysfunction in diabetic cardiomyopathy [21], [22]. However, the mechanism that underlies Serca2 loss in heart disease is not understood.

#### Myofilament proteins

Myosin filaments in the heart function as complexes composed of α and β subunits. The rodent heart expresses three forms of myosin: an α-MHC form, which has the highest ATPase activity and contractile velocity; an α- and β-MHC form; and a β-MHC form, which has the lowest contractile capability [23]. During fetal development, α-MHC replaces β-MHC as the dominant transcript in cardiac muscle [24], and this difference is maintained perinatally. Therefore, a decrease in the α-MHC/β-MHC ratio is used as a marker of fetal gene reactivation in rodent hearts and is associated with cardiac hypertrophy [25]. It should be noted that the regulation of myosins in the human heart is different; while α-MHC predominates in non-failing adult rodent hearts, the adult human ventricle expresses approximately 95% β-MHC [26]–[28].

Other non-myosin cytoskeletal proteins which are changed in the hypertrophied heart include actin and titin. Skeletal α-actin is highly expressed in fetal hearts and is not expressed in the adult heart; instead, adult hearts express cardiac α-actin [29]. Therefore, skeletal α-actin is considered a member of the FGP and is used as a marker of hypertrophy, and is associated with cardiac dysfunction [30]. A similar switch is observed in the expression of titin isoforms: embryonic hearts express much higher levels of the N2BA isoform of titin, which is replaced by the shorter N2B titin isoform in the perinatal and adult heart [31]; therefore, expression of the long-form N2BA in the adult heart is used as a marker of pathological cardiac hypertrophy.

#### Peptide hormones

Atrial and brain natriuretic peptide (ANP and BNP) are small peptide hormones. The prohormone precursors of ANP and BNP are encoded by the *Nppa* and *Nppb* genes, respectively, and are some of the most commonly measured members of the fetal gene program. ANP expression is an early marker of cardiac commitment and is activated by the fetal cardiac transcription factors GATA4 and NKS2-5 [32]. In adult hearts, ANP and BNP are used as markers of heart failure because they are secreted in response to cardiac wall strain [33], although the mechanism of release is only just now being elucidated [34]. While they largely regulate natriuresis and reduce blood pressure, ANP and BNP also antagonize cardiac hypertrophy [35], [36] and fibrosis [37], and stimulate lipolysis [38].

#### Transcription factors

A suite of transcription factors governs the formation of the fetal heart and is used to mark FGP upregulation in the adult heart. The GATA4 transcription factor is expressed at high levels in the fetal myocardium and drives the formation of the fetal heart. It is required for normal valvular development [39], [40], activates a broad group of cardiac-specific genes including ANP [41] and α-MHC [42], and is required for upregulation of β-MHC in pathological hypertrophy after trans-aortic constriction surgery [43].

The NK2 homeobox protein (NKX2-5/CSX) is one of the earliest markers of commitment to the cardiac lineage in embryonic mesoderm [44]. Its expression is confined to the heart, and is upregulated in fetal development and maintained in the postnatal and adult heart [45]. NKX2-5 expression requires GATA4 [44] and overexpression of these two genes in tandem drives commitment of mesenchymal stem cells to the cardiac lineage [46]. Recapitulation of NKX2-5 and associated transcription factors (GATA4, MEF2, and SP1) expression occurs in animal models of congestive heart failure [47], and is considered a marker of fetal gene reactivation.

Collaborating transcription factors that regulate fetal genes through interactions with GATA4 and CSX include MEF2 and Hand1/2. MEF2 governs a family of transcription factors that regulates fetal gene expression in the adult heart. Hand1/2 (eHand/dHand) are expressed at high levels in the fetal heart, and both Hand1 and Hand2 activate *Nppa*, via physical associations with MEF2 [48] and NKX2.5 [49].

Although the relationship between fetal genes and pathological hypertrophy is well characterized [1], [50]–[52], it is not known how these genes are affected by diabetes. Therefore, the purpose of this systematic review was to determine whether the expression of fetal genes is a consistent marker of cardiac hypertrophy in studies that use rodent models of diabetes. We found no correlations between fetal gene expression and the HW:BW index in rodent models of diabetes, and our results show that methods of inducing experimental diabetes significantly affect the expression of fetal genes in rodent hearts.

## Methods

### Inputs

#### Diabetogenics

The search terms for diabetogenic drugs and commonly used animal models of diabetes were generated from a review article [53]. Drug-induced diabetes is most commonly accomplished by injection of cytotoxic glucose analogs, i.e. streptozotocin (STZ) and alloxan, both of which are taken up by glucose transporter 2 into pancreatic β-cells [54]. The diabetogenic drugs that were included in the search parameters were STZ, alloxan, dithizone, or 8-hydroxyquinolone, but it should be noted that only the former two drugs returned results. STZ- and alloxan-induced models of diabetes were categorized as “drug,” spontaneous/genetic models were categorized as “spontaneous,” and diet- or diet/drug-induced models were categorized as “other” for our analyses.

#### Fetal genes

The inputs for the most commonly measured fetal genes were gathered from review articles [1], [51], [52]. Several of these transcription factors were included in the *a priori* article search, including NFAT, SRF, and the SMAD family, but these did not return any additional results when included with our other search parameters.

### Search

**Figure 1:**
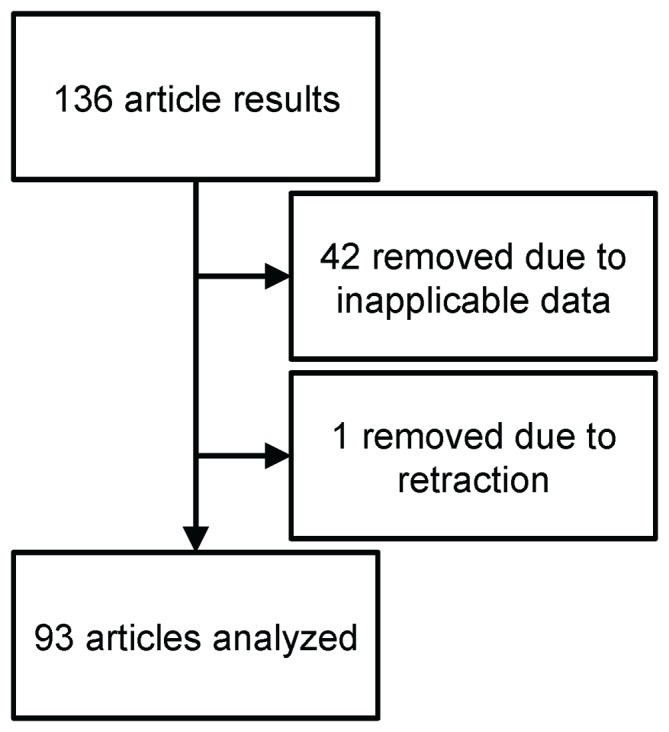
Flowchart of study selection.

A MeSH search using the previously described inputs (**TABLE 1**) was used to generate an a priori list of 135 articles in PubMed. Of the 136 articles returned by the search, 42 were excluded for containing inapplicable data and one was excluded for having been retracted (**FIGURE 1**). Therefore, 93 articles were included in this review (**TABLE S1**).

**Table 1:** Complete search terms used to collect articles from the PubMed database.

(cardiac[MeSH Major Topic] OR cardiac hypertrophy[MeSH Terms] OR cardiomegaly[MeSH Terms] OR Hypertrophy, Left Ventricular[MeSH Terms] OR Cardiomyopathy, Dilated[MeSH Terms] OR diabetic cardiomyopathy[MeSH Terms]) AND
(diabetes[MeSH Major Topic] OR prediabetes[MeSH Terms] OR hyperglycemia[MeSH Terms] OR insulin resistance[MeSH Terms] OR streptozotocin[All Fields] OR alloxan[All Fields] OR Dithizone[All Fields] OR 8-hydroxyquinolone[All Fields] OR spontaneously diabetic[All Fields] OR db/db[All Fields] OR diabetic mouse[All Fields] OR diabetic mice[All Fields] OR diabetic rat*[All Fields] OR non-obese diabetic[tw] OR biobreeding rat[All Fields]) AND
(fetal gene[tw] OR fetal gene program[tw] OR myosin heavy chain[tw] OR N2BA*[tw] OR GATA4[tw] OR NFAT[tw] OR (Csx[tw] OR NKX*[tw]) OR SRF[tw] OR MEF2[tw] OR Hand1/2[tw] OR Smad[tw] OR ANP[tw] OR ANF[tw] OR atrial natriuretic peptide[tw] OR atrial natriuretic factor[tw] OR BNP[tw] OR BNF[tw] OR brain natriuretic peptide[tw] OR brain natriuretic factor[tw] OR alpha skeletal actin[tw] OR skeletal actin[tw] OR alpha actin[tw] OR SERCA*[tw]) AND
Journal Article[PT] AND
(cardiomyocyte[MeSH Terms] OR primary cell*[MeSH Terms] OR ventricular myocyte[tw] OR models, animal[MeSH Terms] OR mouse heart*[MeSH Terms] OR rat heart*[MeSH Terms]) AND
1980:2013[dp]

We categorized diabetogenic categories as follows: “drug-induced” (STZ or alloxan), “spontaneous” genetic models, or “other.” “Other” included diet-induced, or combination diet- and low-dose STZ-induced diabetes. Diabetic phenotype was coded as type 1 diabetes mellitus (T1DM) or type 2 (T2DM) based on whether the intervention produced primary insulin deficiency (e.g. STZ models of diabetes) or insulin resistance (e.g. hyperinsulinemic genetic models of diabetes).

### Statistics

Specific effect sizes were not reported for most studies; therefore, we categorized changes as up (1), down (−1), or no change (0), and used non-parametric Mann-Whitney, Chi-squared, and Spearman regression analyses as appropriate. Significance was set at P<0.05.

## Results

### General description of methods/animal characteristics

Overall, 38 studies (33% of this database) used T2DM models while 78 studies (67%) used T1DM models. By far the most commonly used T1DM model was the Sprague-Dawley rat induced with STZ (**TABLE 2**). The most commonly used model of T2DM was the *db/db* mouse. Only one study used alloxan as their diabetogenic [55] and no studies used dithizone or 8-hydroxyquinolone. Therefore, as alloxan and STZ have very similar mechanisms of action, we grouped these drugs together into a single category of drug-induced diabetes for statistical analyses.

**Table 2:** Descriptive summary of rodent models of experimental diabetes.

Drug	Strain	Species	N	Diabetes type
Alloxan	Sprague-Dawley	Rat	1	T1DM
Spontaneous	Akita	Mouse	3	T1DM
STZ	Sprague-Dawley	Rat	33	T1DM
STZ	Wistar	Rat	16	T1DM
STZ	C57BL/6	Mouse	15	T1DM
STZ	FVB	Mouse	4	T1DM
STZ	Wistar-Kyoto	Rat	2	T1DM
STZ	—	—	2	T1DM
STZ	CR1:W1	Rat	1	T1DM
STZ	CD1	Mouse	1	T1DM
Combination (Diet + STZ)	Wistar	Rat	2	T2DM
Combination (Diet + STZ)	Sprague-Dawley	Rat	1	T2DM
Diet	Wistar	Rat	2	T2DM
Diet	Sprague-Dawley	Rat	1	T2DM
Diet	C57BL/6	Mouse	1	T2DM
Spontaneous	db/db	Mouse	14	T2DM
Spontaneous	ZDF	Rat	5	T2DM
Spontaneous	Goto-Kakizaki	Rat	4	T2DM
Spontaneous	OLETF	Rat	3	T2DM
Spontaneous	NOD	Mouse	2	T2DM
Spontaneous	UCD-T2DM	Rat	1	T2DM
Spontaneous	OVE26	Mouse	1	T2DM
Spontaneous	ob/ob	Mouse	1	T2DM

Dose reporting varied for the studies that used STZ or alloxan. Single-dose STZ was the most common method (n = 58); however, the number of doses varied from two to seven (**TABLE 3**). Two studies did not report the number of doses of the diabetogenic agent.

**Table 3:** Summary of dosing regimens used to induce experimental diabetes in rodents.

Species	Strain	Sex	Diabetogenic	Doses	N	Diabetes type
Mouse	C57BL/6	M	STZ	1	5	T1DM
Mouse	C57BL/6	M	STZ	2	1	T1DM
Mouse	C57BL/6	M	STZ	3	3	T1DM
Mouse	C57BL/6	M	STZ	5	3	T1DM
Mouse	C57BL/6	M	STZ	7	2	T1DM
Mouse	C57BL/6	M and F	STZ	7	1	T1DM
Mouse	CD1	M	STZ	1	1	T1DM
Mouse	FVB	M	STZ	1	1	T1DM
Mouse	FVB	M	STZ	5	3	T1DM
Rat	CR1:W1	Not reported	STZ	1	1	T1DM
Rat	Not reported	F	STZ	1	1	T1DM
Rat	Not reported	M	STZ	1	1	T1DM
Rat	Sprague-Dawley	F	STZ	1	5	T1DM
Rat	Sprague-Dawley	F	STZ	3	1	T1DM
Rat	Sprague-Dawley	M	alloxan	1	1	T1DM
Rat	Sprague-Dawley	M	STZ	1	26	T1DM
Rat	Sprague-Dawley	Not reported	STZ	1	1	T1DM
Rat	Wistar	M	STZ	1	14	T1DM
Rat	Wistar	M	STZ	Not reported	2	T1DM
Rat	Wistar-Kyoto	M	STZ	1	2	T1DM
Rat	Sprague-Dawley	F	High fructose diet + STZ	1	1	T2DM
Rat	Wistar	M	High fructose/high sugar diet + STZ	1	2	T2DM

One study used both male and female C57BL/6 mice for their project [56], 16 used female mice and rats, and 96 used male mice and rats. Ninety-six studies reported either a starting age or weight. Twenty studies did not report the age of their experimental animals. Thirty-three did not report the change in body weight of their animals (i.e. wasting, fat, or no change) after diabetogenic intervention.

Heart weight (HW) was the most frequent method of reporting cardiac hypertrophy (**TABLE 4**). The most common indexing method was normalization to body weight; few studies reported the HW:tibia length (TL) index. Although 33 studies did not report total body weight, seven of these 33 studies reported the HW:BW or HW:TL index.

**Table 4:** Frequency of methods used to report cardiac hypertrophy.

Measure	N
HW	48
HW:BW	43
LVW:BW	8
Cardiomyocyte area	8
HW:TL	7
LVW	7
RVW	1
RVW:BW	1

Measure indicates the value or index used to report cardiac hypertrophy. N indicates the number of studies that reported that measure. HW = heart weight; BW = body weight; TL = tibia length; LVW = left ventricle weight; RVW = right ventricle weight.

### The type of diabetes/diabetogenic moderates hypertrophy

Neither the age of animals at sacrifice (stratified in 5-week increments) or animal sex had any effect on absolute final heart weight. Rodent species did not affect absolute final HW; however, diabetic rats were significantly more likely to show an increase in HW:BW compared to diabetic mice (P<0.05). This finding may be confounded by the fact that diabetic rats were significantly more likely to show loss of body weight compared to diabetic mice (P<0.05).

**Figure 2:**
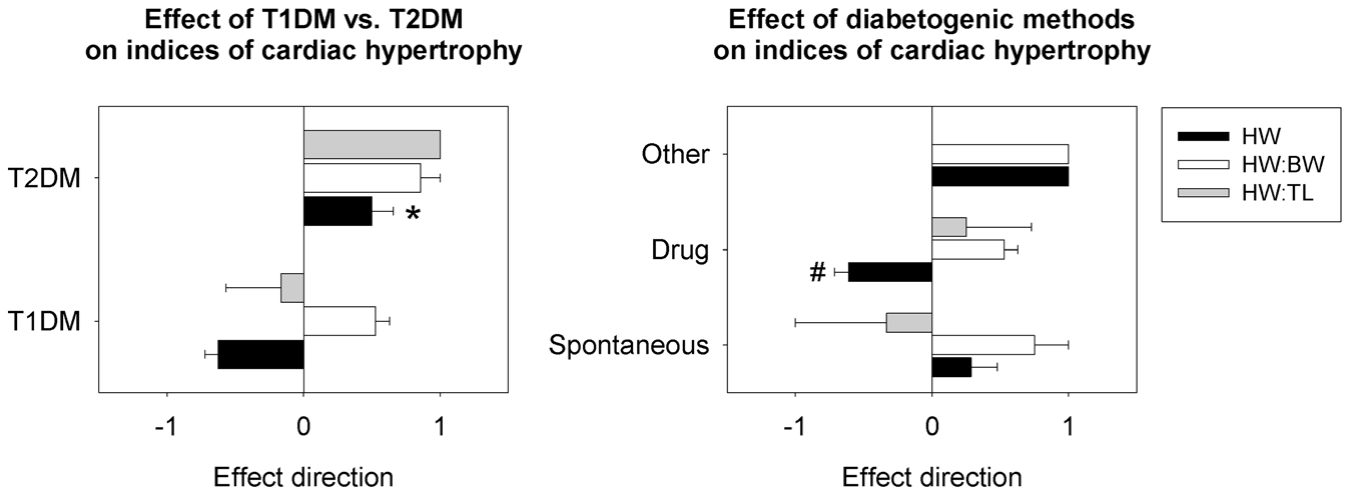
Effect of diabetes type and methods of diabetogenicity on indices of cardiac hypertrophy in experimental rodent models of diabetes. The type of diabetes and the method of inducing diabetes significantly affect absolute heart weight but not the HW:BW index in rodent models of diabetes. * significant effect of diabetes type; # significant effect of diabetogenic from both other categories. Significance set at P<0.05. T1DM  =  type 1 diabetes mellitus; T2DM  =  type 2 diabetes mellitus; HW  =  heart weight; BW  =  body weight; TL  =  tibia length.

The difference in absolute heart weight from controls was significantly lower in T1DM models than T2DM models (P<0.05) (**FIGURE 2**). The type of diabetes had no significant effect on HW:BW, left ventricular weight:body weight, cardiomyocyte area, or the presence of cardiac dysfunction. The category of diabetogenic agent (drug vs. spontaneous vs. other) had similar effects on hypertrophy. Diabetogenic category significantly influenced absolute heart weight (P<0.05), but had no significant effect on HW:BW or HW:TL.

**Figure 3:**
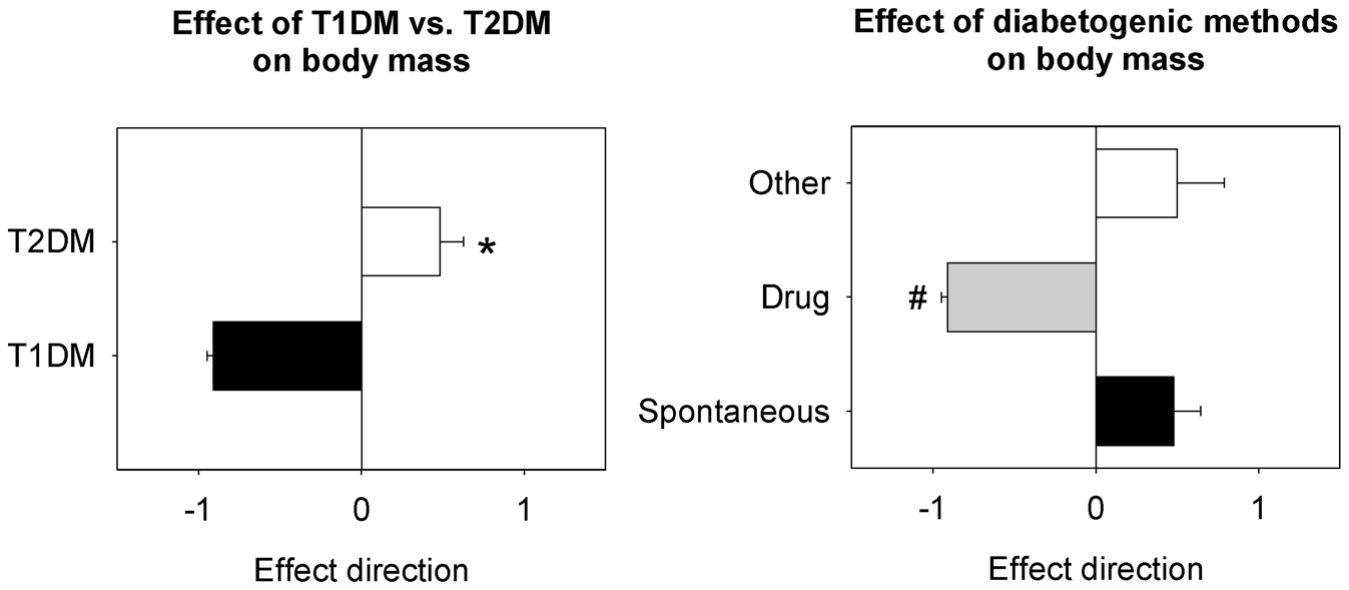
Effect of diabetes type and methods of diabetogenicity on body weight in experimental rodent models of diabetes. The type of diabetes and the method of inducing diabetes significantly affect final body weight in rodent models of diabetes. * significant effect of diabetes type; # significant effect of diabetogenic from both other categories. Significance set at P<0.05. T1DM  =  type 1 diabetes mellitus; T2DM  =  type 2 diabetes mellitus.

Because the HW:BW ratio was the most common method of indexing, we also investigated the effects of diabetes type and diabetogenic on body weight (**FIGURE 3**). T1DM animals showed significant loss of body weight relative to T2DM animals (P<0.05). Drug-induced diabetic animals also showed significant loss of body weight compared to spontaneous- or diet/drug-induced models of diabetes (P<0.05).

### The effect of diabetes duration on cardiac hypertrophy

We coded the duration of diabetes as the time from the final drug administration to the time the animals were sacrificed (for drug-induced diabetes), or as the amount of time a diet was consumed (for diet-induced diabetes). We then stratified the time of the intervention in 5-week increments ranging from 0 to >20 weeks. The HW and the HW:BW index were not affected by rodent age at the start of the intervention, rodent age at the time of analysis, or the duration of the diabetogenic intervention.

There were not sufficient data in each 5-week increment to compare effects of time on fetal genes. Therefore, we re-categorized the shortest duration of diabetes (0–5 weeks) as “acute” and pooled the longer durations as “chronic” (range: 5.1–32 weeks). Acute vs. chronic diabetes had no effect on absolute final heart weight, or body weight. However, increased HW:BW was significantly more common in chronic models than acute models. Acute vs. chronic diabetes had no effect on any fetal gene protein or mRNA levels.

Since some studies used multiple rather than single STZ dosing, we controlled for effects of dose, and interactions of dose by duration of diabetes. Dose number (ranging from 1–7) had no effect on HW or the HW:BW index. There were not a sufficient number of studies in each dose category to compare the effect of dose on fetal gene protein or mRNA levels.

### Fetal genes are not correlated with HW:BW or other fetal genes

#### Serca2

**Figure 4:**
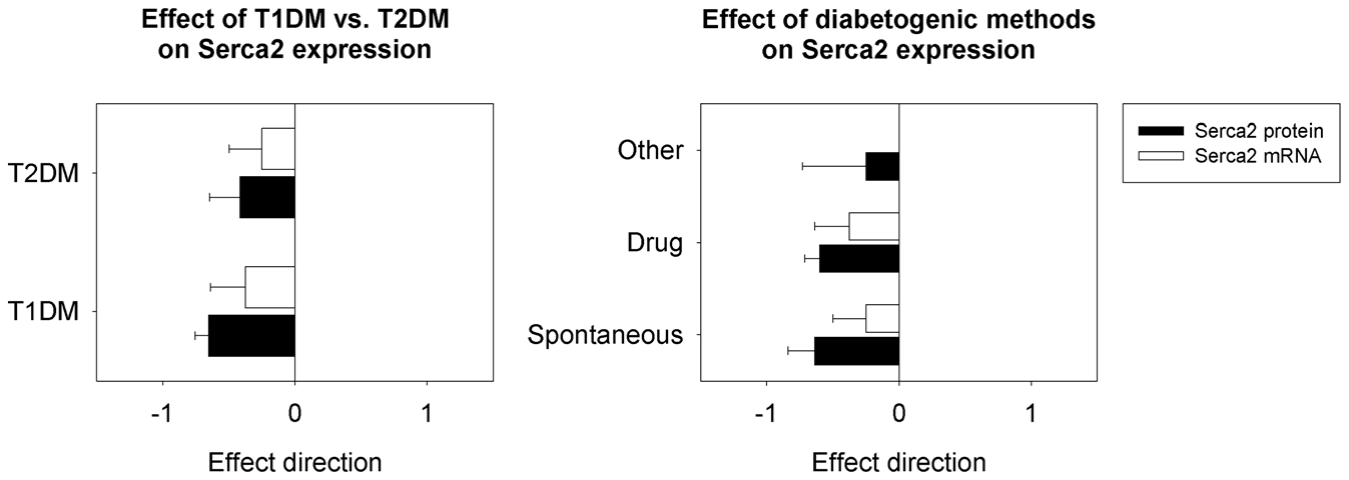
Effect of diabetes type and methods of diabetogenicity on cardiac Serca2 expression. Serca2 is not affected by either diabetes type or by different methods of inducing diabetes in the hearts of experimental rodent models of diabetes. T1DM  =  type 1 diabetes mellitus; T2DM  =  type 2 diabetes mellitus.

Neither the diabetogenic category nor the diabetes type had any significant effect on Serca2 protein or mRNA levels. Overall, diabetic animals consistently showed lower Serca2 protein levels relative to non-diabetic controls (**FIGURE 4**). There was no significant effect of species (mouse vs. rat) on Serca2 expression, and no interaction of species with diabetogenic method.

#### Myofilament isoforms

**Figure 5:**
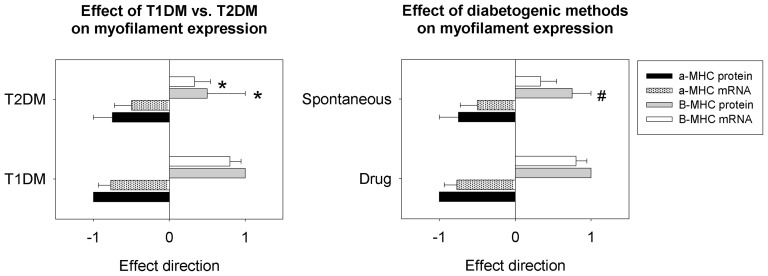
Effect of diabetes type and methods of diabetogenicity on cardiac myosin expression. The type of diabetes and the method of inducing diabetes significantly affect the expression of myosins in the hearts of experimental rodent models of diabetes. * significant effect of diabetes type; # significant effect of diabetogenic category. Significance set at P<0.05. T1DM  =  type 1 diabetes mellitus; T2DM  =  type 2 diabetes mellitus.

The type of diabetes had no effect on α-MHC protein or mRNA levels. However, the type of diabetes moderated the expression of β-MHC. Although protein and transcript levels of β-MHC were upregulated in diabetic animals relative to controls overall, the extent of this upregulation was significantly greater in the T1DM group compared to the T2DM group (P<0.05) (**FIGURE 5**).

Protein and mRNA levels of α-MHC and protein levels of β-MHC were not different between diabetogenic groups. mRNA levels of β-MHC were significantly higher in the drug-induced diabetogenic category compared to the spontaneous diabetogenic category (P<0.05). Interestingly, rodent species (mouse vs. rat) had a significant effect on β-MHC mRNA levels; rats more frequently showed an increase in β-MHC mRNA compared to mouse models (P<0.05), but there was no significant interaction of species with diabetogenic method.

There were not sufficient data to compare the α-MHC/β-MHC ratio between groups, and the small numbers of studies in our dataset showed conflicting results. The ratio decreased in a spontaneous rat model of T2DM [21] and in a rat model of STZ-induced T1DM [57]. However, it increased in the hearts of female type 2 diabetic *db/db* mice [12].

#### Natriuretic peptides

**Figure 6:**
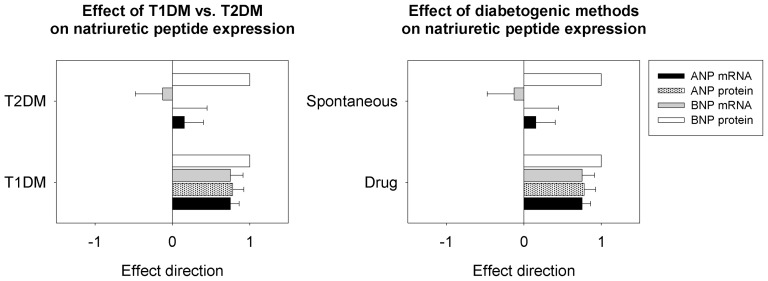
Effect of diabetes type and methods of diabetogenicity on cardiac natriuretic peptide expression. The type of diabetes and the method of inducing diabetes significantly affect the expression of natriuretic peptides in the hearts of experimental rodent models of diabetes. T1DM  =  type 1 diabetes mellitus; T2DM  =  type 2 diabetes mellitus.

Animal models of T1DM tended to show higher transcript levels of ANP relative to controls than T2DM models (P = 0.057) (**FIGURE 6**). The diabetogenic category did not have any effect on ANP protein levels; however, drug-induced models also tended to show higher ANP transcript levels relative to spontaneous models (P = 0.057). Diabetogenic category did not have any effect on BNP mRNA, and there were not sufficient data to compare BNP protein levels between diabetogenic categories. However, our dataset included three studies that showed increased BNP protein levels in the hearts of type 1 diabetic Akita mice [58], type 1 diabetic STZ-induced diabetic FVB mice [59], and UC Davis type 2 diabetic rats [60]. There was no significant effect of species (mouse vs. rat) on natriuretic peptide expression, and no interaction of species with diabetogenic method.

#### Correlations of fetal genes with cardiac hypertrophy

Spearman regression was performed to correlate fetal gene mRNA and protein levels with absolute heart weight and the HW:BW index. We report correlations for which N≥3 studies.

ANP protein levels were negatively correlated with absolute heart weight (P<0.05), but mRNA levels of ANP were not associated with heart weight (**TABLE 5**). Similarly, α-MHC protein was directly correlated with increases in absolute heart weight (P<0.05), but transcript levels of α-MHC were not.

**Table 5:** Spearman correlations of fetal genes with absolute heart weight.

Correlate	ρ	P	N	P<0.05
ANP protein	−0.730	0.03	8	*
ANP mRNA	−0.003	0.99	15	NS
BNP mRNA	0.354	0.27	11	NS
Serca2 protein	0.170	0.60	11	NS
Serca2 mRNA	−0.411	0.23	10	NS
α-MHC protein	1.000	0.02	5	*
α-MHC mRNA	0.362	0.29	10	NS
β-MHC protein	−1.000	0.08	4	NS
β-MHC mRNA	−0.501	0.09	12	NS

Spearman correlation coefficients; ρ =  correlation coefficient, P = p value, N = # studies.

The HW:BW index did not correlate with the expression of any fetal genes (**TABLE 6**). There were not enough data to correlate fetal genes with the HW:TL index, as only seven studies reported this index.

**Table 6:** Spearman correlations of fetal genes with the HW:BW index.

Correlate	ρ	P	N	P<0.05
ANP protein	0.408	0.45	5	NS
ANP mRNA	0.000	0.98	9	NS
BNP mRNA	0.612	0.23	5	NS
Serca2 protein	0.234	0.39	15	NS
Serca2 mRNA	0.310	0.56	6	NS
α-MHC protein	−0.250	0.68	5	NS
α-MHC mRNA	0.452	0.23	8	NS
β-MHC mRNA	−0.186	0.58	10	NS

Spearman correlation coefficients; ρ =  correlation coefficient, P = p value, N = # studies.

We then examined these correlations separately within type 1 and type 2 diabetes models. In T1DM models, no fetal genes correlated with heart weight or the HW:BW index (**TABLE 7** **,** **8**). However, in T2DM models, BNP mRNA levels were directly correlated to absolute heart weight (P<0.05) (**TABLE 9**), although protein levels of BNP were not.

**Table 7:** Spearman correlations of fetal genes with absolute heart weight in rodent models of type 1 diabetes.

Correlate	ρ	P	N	P<0.05
ANP protein	−0.707	0.14	6	NS
ANP mRNA	−0.167	0.66	7	NS
BNP mRNA	−0.577	0.42	4	NS
Serca2 protein	−0.250	0.49	9	NS
Serca2 mRNA	−0.816	0.08	4	NS
α-MHC protein	1.000	0.08	4	NS
α-MHC mRNA	0.632	0.18	6	NS
β-MHC protein	1.000	0.33	3	NS
β-MHC mRNA	−0.571	0.12	8	NS

Spearman correlation coefficients; ρ =  correlation coefficient, P = p value, N = # studies.

**Table 8:** Spearman correlations of fetal genes with the HW:BW index in rodent models of type 1 diabetes.

Correlate	ρ	P	N	P<0.05
ANP protein	0.408	0.45	5	NS
ANP mRNA	0.123	0.75	8	NS
BNP mRNA	0.500	1.00	3	NS
Serca2 protein	0.200	0.51	12	NS
Serca2 mRNA	0.395	0.52	5	NS
α-MHC mRNA	0.452	0.23	8	NS
β-MHC protein	1.000	0.33	3	NS
β-MHC mRNA	−0.186	0.58	10	NS

Spearman correlation coefficients; ρ =  correlation coefficient, P = p value, N = # studies.

**Table 9:** Spearman correlations of fetal genes with absolute heart weight in rodent models of type 2 diabetes.

Correlate	ρ	P	N	P<0.05
ANP mRNA	0.559	0.14	8	NS
BNP mRNA	0.833	0.01	7	*
Serca2 mRNA	0.000	1.00	6	NS
α-MHC mRNA	−0.577	0.42	4	NS
β-MHC mRNA	0.333	0.75	4	NS

Spearman correlation coefficients; ρ =  correlation coefficient, P = p value, N = # studies.

#### Correlations of fetal genes with each other

In the 12 studies that measured both ANP and BNP, ANP protein correlated with BNP mRNA (P<0.05). The eight studies that measured Serca2 protein and Serca2 mRNA showed a direct correlation between these two (P<0.05). Finally, in nine studies α-MHC protein was negatively correlated with β-MHC protein (P<0.05). However, there were no correlations between fetal genes in different categories: the expression of fetal myofilaments did not correlate with Serca2 or the natriuretic peptides, and vice versa.

### Miscellaneous results

Several of our search terms returned too few studies for statistical analysis; these results are summarized below.

#### Transcription factors

Two studies showed that NCX levels decreased in the hearts of STZ-induced diabetic rats [61], [62]. However, cardiac NCX protein was unchanged in another study with STZ-induced diabetic rats [63], and was increased in the Akita mouse [64]. One study showed that cardiac MEF2 was reduced in STZ-induced diabetic rats [57], but another study showed an increase [65]. Cardiac mRNA levels of MEF2 were increased in STZ-induced diabetic mice [66]. E-hand and D-hand protein levels were decreased in the hearts of STZ-induced rats [57], but did not change in another study with STZ-induced diabetic rats [65].

Both SMAD2 [67] and SMAD7 [68] protein levels were increased in STZ-induced diabetic rat hearts. Cardiac phospho-SMAD2 and phospho-SMAD3 were also increased in high-fructose-fed diabetic rats [69] and STZ-induced diabetic rats [70]. One study found an increase in phosphorylated GATA4 in STZ-induced diabetic rat hearts [71].

#### Cardiac α-actin

Cardiac α-actin is the form of actin expressed in the postnatal and adult heart; downregulation of cardiac α-actin is indicative of fetal gene activation in cardiomyocytes. In our database, one study showed that transcript levels of cardiac α-actin did not change in STZ-induced rats [14]. Two studies showed that cardiac α-actin was reduced in STZ-induced diabetic rats [72] and *db/db* type 2 diabetic mice [73].

#### Natriuretic peptides

One study showed that ANP protein was reduced in the atria but increased in the ventricles of STZ-induced diabetic rats [74]. Insulin normalized an increase in left-ventricular transcript levels of ANP in STZ-induced diabetic rats in one study [15]. Both plasma ANP and granular ANP within cardiomyocytes were increased in high-fructose fed C57BL/6 mice [75]. Finally, the mRNA levels of the natriuretic peptide receptors NPR-A and NPR-B were increased in STZ-induced C57BL/6 mouse hearts [76].

## Discussion

Results of this study show that diabetogenic methods affect the development of cardiac hypertrophy in diabetic animals, and the expression of fetal genes. Models of T2DM showed cardiac hypertrophy relative to controls, while models of T1DM showed significant loss of heart weight. None of the cardiac fetal genes analyzed in this study correlated with the HW:BW index, the most commonly reported estimate of cardiac hypertrophy. The only members of the FGP that were associated with absolute heart weight were α-MHC protein and ANP protein, which had significant positive and negative correlations with heart weight, respectively. However, the mRNA levels of α-MHC and ANP did not correlate with heart weight. When we separated this analysis by the type of diabetes, BNP mRNA levels were significantly positively correlated with heart weight in type 2 models. Results of this analysis suggest that fetal genes are not generally correlates of cardiac hypertrophy in animal models of diabetes, and that fetal gene expression is confounded by animal species, the type of diabetes (type 1 vs. type 2), and the method of inducing experimental diabetes.

We also analyzed the correlations of fetal genes with each other to determine whether they showed similar patterns of expression. Interestingly, there were absolutely no correlations in the expression of fetal genes from different functional categories. The expression of genes with similar functions showed some agreement: for example, ANP protein was directly correlated with BNP mRNA levels, Serca2 mRNA and protein levels correlated with each other, and levels of α-MHC and β-MHC protein were negatively correlated. These data suggest that fetal genes within similar functional categories, e.g. the natriuretic peptides or the heavy chain myosins, may be co-regulated in diabetic hearts. However, the natriuretic peptides did not correlate with Serca2 or the myosins, and vice versa. Collectively, these findings do not support the concept of a cohesively regulated FGP in the diabetic heart.

Most studies reported that cardiac expression of β-MHC was increased in experimental diabetic animals relative to controls. However, this was significantly moderated by the type of diabetes: type 1 animals consistently showed greater upregulation of β-MHC protein and mRNA than type 2 models. β-MHC was also not associated with cardiac hypertrophy in our correlation analysis. Surprisingly, the expression of β-MHC mRNA was significantly higher in rats compared to mice.These results suggest that changes in fetal myofilament isoforms in the adult heart do not strictly indicate cardiac hypertrophy, and are confounded by animal species. It has already been proposed, for example, that β-MHC is a more specific marker of fibrosis than hypertrophy [77]. However, this does not explain the discordant results we found regarding the α-MHC/β-MHC ratio in diabetic hearts. Studies reported that this ratio decreased in spontaneously type 2 diabetic rats [21] and in STZ-induced type 1 diabetic rats [57], but increased n the hearts of female type 2 diabetic *db/db* mice [12].

### Hypertrophic phenotyping methods

We found that methods of reporting hypertrophy significantly influence the interpretation of the cardiac phenotype. Absolute heart weight was the most common method of reporting hypertrophy, followed by the HW:BW index. Only seven studies reported the HW:TL index, which is a more reliable correlate of cardiomyocyte area than either HW or HW:BW [78]. Importantly, according to the HW:BW index, there were no significant differences in hypertrophy between animal models of diabetes, and all animals showed hypertrophy. However, both absolute heart weight and body weight were significantly different between type 1 and type 2 animals: type 1 animals generally showed cachexia and a loss of heart weight, and type 2 models showed obesity and an increase in heart weight. We found that seven studies did not report body weight and only reported either the HW:BW or HW:TL ratio. Until more is understood about hypertrophy in various animal models of diabetes, we suggest that studies report multiple indices of hypertrophy, because the results of this analysis show that simple indexing methods can mask important phenotypic differences.

### Diabetogenic methods

We propose that the use of toxic glucose analogues for inducing diabetes, which is one of the most common methods for studying diabetic cardiomyopathy at the present time, should be reexamined. At high doses, these diabetogenics induce a model of T1DM that develops cardiac atrophy, cachexia, and primary insulin deficiency. This is a clear departure from the phenotype of humans who develop diabetic cardiomyopathy secondary to T2DM, who are typically hyperinsulinemic, obese, insulin resistant, and show cardiac hypertrophy. The incidence of human diabetes is also overwhelmingly type 2 (approximately 95% of all diabetics); therefore, models of primary insulin deficiency induced with toxic glucose analogues have limited application to the clinical entity of diabetic cardiomyopathy.

These diabetogenic agents also may be toxic to multiple organs and have independent effects on cardiac function. The mechanism of action of STZ and alloxan is inducing cell death secondary to alkylating and oxidative DNA damage, and disruption in calcium kinetics [79]. Other mechanisms of β-cell toxicity include inhibition of O-linked β-N-acetylglucosaminidase, which removes O-linked β-N-acetylglucosamine (O-GlcNAc) groups from serine/threonine residues of proteins [80]. This toxicity is relatively selective to pancreatic β-cells; however, animals treated with alloxan or STZ also exhibit hepatoxocity and signs of kidney damage [81]. STZ also independently reduces cardiomyocyte contractility [82] and both alloxan and STZ induce cardiomyocyte dysfunction [83], [84].

Many hypotheses have been proposed for the dysfunctional phenotype of the diabetic heart, including disruption in calcium kinetics, increased oxidative stress, energetic disturbances due to glucotoxicity and/or lipotoxicity, inflammation, and cardiomyocyte apoptosis; for recent reviews, see [85] and [86]. It is critical to recognize that the independent effects of toxic glucose analogs on these aspects of cardiomyocyte function that are considered indicative of diabetic cardiomyopathy, such as lipotoxicity and oxidative stress, have simply not been examined. Therefore, the use of toxic glucose analogues may not produce a physiologically relevant model of diabetic cardiomyopathy.

### Interpretations and proposed mechanisms

The basic relationship between cardiac hypertrophy and fetal gene activation is unresolved. Although many excellent reviews have been published on the transcriptional mechanisms that regulate these genes [2], [50]–[52], [87], the pathways that activate these mechanisms are very poorly understood. It is also not yet established whether FGP activation is a cause or a result of hypertrophy, or whether it is beneficial or decompensatory. For example, the natriuretic peptides antagonize hypertrophy and fibrosis [35], [88], [89], suggesting that their action is beneficial. Conversely, the loss of Serca2 expression in lieu of fetal-type calcium handling proteins is clearly detrimental for the adult heart [18], [21], [62], [90]–[92].

An emerging hypothesis proposes that fetal gene expression in the adult heart represents compensatory dedifferentiation, or fetal “reprogramming,” of adult cardiomyocytes. Adult cardiomyocytes show considerable plasticity in their differentiation state [93], and dedifferentiate in response to insults such as myocardial infarction, chronic hypertension, and heart failure [94], [95]. However, the underlying mechanisms are not well understood, and cardiomyocyte plasticity may even be intrinsically different between animal strains [96]. Indeed, the ability to revert from an adult phenotype may be an inherently protective process in the adult heart [95], mimicking the highly cardioprotective phenotype of the fetal heart [97]. This has led to speculation that fetal gene activation in pathological hypertrophy is a protective mechanism [2], [50] and is supported by evidence that the expression of a fetal transcriptome is highly cardioprotective [97], [98]. However, this hypothesis does not yet explain why some fetal genes are regulated differently in fetal vs. diseased hearts. For example, it is not well established why the *Nppa* gene is reactivated in heart disease [99], and *Nppa* has distinct regulatory sequences that are activated in the embryonic heart and the adult failing heart [100]. The question is complicated by the fact that the fetal and failing hearts are not the only ones that express fetal genes; an adult heart deprived of afterload also upregulates fetal gene expression [101].

It is also possible that the expression of fetal genes is closely tied to myocardial metabolism. This would explain why the presence of simultaneous metabolic disease and cardiac hypertrophy would have confounding effects on fetal gene expression in diabetic hearts. The adult heart upregulates glycolytic metabolism during pathological hypertrophy, and it has been proposed that this shift toward fetal-like myocardial metabolism underlies fetal gene activation and cardiac dysfunction [86], [102]. The diabetic heart, by contrast, becomes almost exclusively reliant on fatty acid oxidation [103], [104]. Therefore, although the diabetic heart develops pathological hypertrophy, the fundamental metabolic differences between the pathologically hypertrophied and diabetic heart may confound the expression of fetal genes in diabetic hearts.

### Limitations

The specific nature of this systematic review limited the number of results returned by our search parameters. For example, our database did not return a sufficient number of studies to correlate the expression of fetal genes with HW:TL, since this was an uncommon method of measuring cardiac hypertrophy. There were also not sufficient data to compare the α-MHC/β-MHC ratio between groups, or to compare changes in BNP protein between drug-induced and spontaneous models of diabetes. Therefore, the specific nature of our search parameters and the small number of studies it returned limits our conclusions regarding these variables.

The studies we examined also included a wide variety of strains and genetic backgrounds (**TABLE 2**). While we were able to detect some significant effects of species on the HW:BW ratio, BW, and β-MHC mRNA, there were not enough studies to compare the effects of strain within species. Therefore, additional studies are needed to determine whether genetic backgrounds influence these parameters in mice and rats.

Finally, we found that chronic models of diabetes were more likely to show increases in HW:BW. Importantly, however, there was no interaction of diabetes duration and the number of doses of toxic glucose analogues. These data suggest that cardiac gene regulation in diabetes is not the same between mice and rats, and that animal models of diabetes show progressive changes in cardiac hypertrophy independent of the dosing regimen.

## Conclusions

In rodent models of diabetes, α-MHC protein and ANP protein levels correlate positively and negatively, respectively, with heart weight. The type of diabetes and the method of diabetogenicity independently moderate body weight and cardiac weight, and the expression of β-MHC. We found absolutely no correlations between fetal genes and the HW:BW index in animal models of diabetes. These findings indicate that fetal genes are not specific markers of hypertrophy in rodent models of diabetes. In addition, this review finds wide variation in current methods of diabetogenicity as well as methods of reporting cardiac hypertrophy. We suggest that studies using experimental rodent models of diabetes report multiple indices of cardiac hypertrophy to improve the quality of research in this field.

## Supporting Information

Table S1 Complete list of references.
(DOCX)
