# Supplementary material for: A Systematic Review of Fetal Genes as Biomarkers of Cardiac Hypertrophy in Rodent Models of Diabetes
Source: PLoS One. 2014 Mar 24;9(3):e92903. doi: 10.1371/journal.pone.0092903 (PMC3963983; doi:10.1371/journal.pone.0092903)
Supplement: Table S1 — Complete list of references. (DOCX) [file pone.0092903.s001.docx]

SUPPLEMENTARY TABLES

**Table S1. Complete list of references**

| [[1](#_ENREF_1)] |
| --- |
| [[2](#_ENREF_2)] |
| [[3](#_ENREF_3)] |
| [[4](#_ENREF_4)] |
| [[5](#_ENREF_5)] |
| [[6](#_ENREF_6)] |
| [[7](#_ENREF_7)] |
| [[8](#_ENREF_8)] |
| [[9](#_ENREF_9)] |
| [[10](#_ENREF_10)] |
| [[11](#_ENREF_11)] |
| [[12](#_ENREF_12)] |
| [[13](#_ENREF_13)] |
| [[14](#_ENREF_14)] |
| [[15](#_ENREF_15)] |
| [[16](#_ENREF_16)] |
| [[17](#_ENREF_17)] |
| [[18](#_ENREF_18)] |
| [[19](#_ENREF_19)] |
| [[20](#_ENREF_20)] |
| [[21](#_ENREF_21)] |
| [[22](#_ENREF_22)] |
| [[23](#_ENREF_23)] |
| [[24](#_ENREF_24)] |
| [[25](#_ENREF_25)] |
| [[26](#_ENREF_26)] |
| [[26](#_ENREF_26)] |
| [[27](#_ENREF_27)] |
| [[28](#_ENREF_28)] |
| [[29](#_ENREF_29)] |
| [[30](#_ENREF_30)] |
| [[31](#_ENREF_31)] |
| [[32](#_ENREF_32)] |
| [[33](#_ENREF_33)] |
| [[34](#_ENREF_34)] |
| [[35](#_ENREF_35)] |
| [[36](#_ENREF_36)] |
| [[37](#_ENREF_37)] |
| [[38](#_ENREF_38)] |
| [[39](#_ENREF_39)] |
| [[40](#_ENREF_40)] |
| [[41](#_ENREF_41)] |
| [[42](#_ENREF_42)] |
| [[43](#_ENREF_43)] |
| [[44](#_ENREF_44)] |
| [[45](#_ENREF_45)] |
| [[46](#_ENREF_46)] |
| [[46](#_ENREF_46)] |
| [[47](#_ENREF_47)] |
| [[48](#_ENREF_48)] |
| [[49](#_ENREF_49)] |
| [[50](#_ENREF_50)] |
| [[51](#_ENREF_51)] |
| [[52](#_ENREF_52)] |
| [[53](#_ENREF_53)] |
| [[54](#_ENREF_54)] |
| [[55](#_ENREF_55)] |
| [[56](#_ENREF_56)] |
| [[57](#_ENREF_57)] |
| [[58](#_ENREF_58)] |
| [[59](#_ENREF_59)] |
| [[60](#_ENREF_60)] |
| [[61](#_ENREF_61)] |
| [[62](#_ENREF_62)] |
| [[62](#_ENREF_62)] |
| [[63](#_ENREF_63)] |
| [[64](#_ENREF_64)] |
| [[65](#_ENREF_65)] |
| [[66](#_ENREF_66)] |
| [[67](#_ENREF_67)] |
| [[68](#_ENREF_68)] |
| [[69](#_ENREF_69)] |
| [[70](#_ENREF_70)] |
| [[71](#_ENREF_71)] |
| [[72](#_ENREF_72)] |
| [[73](#_ENREF_73)] |
| [[74](#_ENREF_74)] |
| [[75](#_ENREF_75)] |
| [[76](#_ENREF_76)] |
| [[77](#_ENREF_77)] |
| [[78](#_ENREF_78)] |
| [[79](#_ENREF_79)] |
| [[80](#_ENREF_80)] |
| [[81](#_ENREF_81)] |
| [[82](#_ENREF_82)] |
| [[83](#_ENREF_83)] |
| [[84](#_ENREF_84)] |
| [[85](#_ENREF_85)] |
| [[86](#_ENREF_86)] |
| [[87](#_ENREF_87)] |
| [[87](#_ENREF_87)] |
| [[88](#_ENREF_88)] |
| [[89](#_ENREF_89)] |
| [[90](#_ENREF_90)] |
| [[91](#_ENREF_91)] |
| [[92](#_ENREF_92)] |

1. Abe T, Ohga Y, Tabayashi N, Kobayashi S, Sakata S, et al. (2002) Left ventricular diastolic dysfunction in type 2 diabetes mellitus model rats. Am J Physiol Heart Circ Physiol 282: H138-148.

2. Aragno M, Mastrocola R, Medana C, Catalano MG, Vercellinatto I, et al. (2006) Oxidative stress-dependent impairment of cardiac-specific transcription factors in experimental diabetes. Endocrinology 147: 5967-5974.

3. Bai SZ, Sun J, Wu H, Zhang N, Li HX, et al. (2012) Decrease in calcium-sensing receptor in the progress of diabetic cardiomyopathy. Diabetes research and clinical practice 95: 378-385.

4. Bai Y, Cui W, Xin Y, Miao X, Barati MT, et al. (2013) Prevention by sulforaphane of diabetic cardiomyopathy is associated with up-regulation of Nrf2 expression and transcription activation. Journal of molecular and cellular cardiology 57: 82-95.

5. Bartels ED, Nielsen JM, Bisgaard LS, Goetze JP and Nielsen LB (2010) Decreased expression of natriuretic peptides associated with lipid accumulation in cardiac ventricle of obese mice. Endocrinology 151: 5218-5225.

6. Basu R, Oudit GY, Wang X, Zhang L, Ussher JR, et al. (2009) Type 1 diabetic cardiomyopathy in the Akita (Ins2WT/C96Y) mouse model is characterized by lipotoxicity and diastolic dysfunction with preserved systolic function. Am J Physiol Heart Circ Physiol 297: H2096-2108.

7. Bidasee KR, Zhang Y, Shao CH, Wang M, Patel KP, et al. (2004) Diabetes increases formation of advanced glycation end products on Sarco(endo)plasmic reticulum Ca2+-ATPase. Diabetes 53: 463-473.

8. Buchanan J, Mazumder PK, Hu P, Chakrabarti G, Roberts MW, et al. (2005) Reduced cardiac efficiency and altered substrate metabolism precedes the onset of hyperglycemia and contractile dysfunction in two mouse models of insulin resistance and obesity. Endocrinology 146: 5341-5349.

9. Bupha-Intr T, Oo YW and Wattanapermpool J (2011) Increased myocardial stiffness with maintenance of length-dependent calcium activation by female sex hormones in diabetic rats. Am J Physiol Heart Circ Physiol 300: H1661-1668.

10. Candido R, Forbes JM, Thomas MC, Thallas V, Dean RG, et al. (2003) A breaker of advanced glycation end products attenuates diabetes-induced myocardial structural changes. Circ Res 92: 785-792.

11. Castoldi G, di Gioia CR, Bombardi C, Perego C, Perego L, et al. (2010) Prevention of myocardial fibrosis by N-acetyl-seryl-aspartyl-lysyl-proline in diabetic rats. Clin Sci (Lond) 118: 211-220.

12. Cheng YS, Dai DZ, Ji H, Zhang Q and Dai Y (2011) Sildenafil and FDP-Sr attenuate diabetic cardiomyopathy by suppressing abnormal expression of myocardial CASQ2, FKBP12.6, and SERCA2a in rats. Acta pharmacologica Sinica 32: 441-448.

13. Christoffersen C, Bartels ED and Nielsen LB (2006) Heart specific up-regulation of genes for B-type and C-type natriuretic peptide receptors in diabetic mice. European journal of clinical investigation 36: 69-75.

14. Connelly KA, Kelly DJ, Zhang Y, Prior DL, Martin J, et al. (2007) Functional, structural and molecular aspects of diastolic heart failure in the diabetic (mRen-2)27 rat. Cardiovasc Res 76: 280-291.

15. Costa MV, Fernandes-Santos C, Faria Tda S, Aguila MB and Mandarim-de-Lacerda CA (2012) Diets rich in saturated fat and/or salt differentially modulate atrial natriuretic peptide and renin expression in C57BL/6 mice. European journal of nutrition 51: 89-96.

16. Daniels A, van Bilsen M, Janssen BJ, Brouns AE, Cleutjens JP, et al. (2010) Impaired cardiac functional reserve in type 2 diabetic db/db mice is associated with metabolic, but not structural, remodelling. Acta physiologica 200: 11-22.

17. D'Souza A, Howarth FC, Yanni J, Dobryznski H, Boyett MR, et al. (2011) Left ventricle structural remodelling in the prediabetic Goto-Kakizaki rat. Experimental physiology 96: 875-888.

18. Essop MF, Chan WA and Hattingh S (2011) Proteomic analysis of mitochondrial proteins in a mouse model of type 2 diabetes. Cardiovascular journal of Africa 22: 175-178.

19. Feng B, Chen S, George B, Feng Q and Chakrabarti S (2010) miR133a regulates cardiomyocyte hypertrophy in diabetes. Diabetes/metabolism research and reviews 26: 40-49.

20. Fredersdorf S, Thumann C, Zimmermann WH, Vetter R, Graf T, et al. (2012) Increased myocardial SERCA expression in early type 2 diabetes mellitus is insulin dependent: In vivo and in vitro data. Cardiovascular diabetology 11: 57.

21. Golfman LS, Wilson CR, Sharma S, Burgmaier M, Young ME, et al. (2005) Activation of PPARgamma enhances myocardial glucose oxidation and improves contractile function in isolated working hearts of ZDF rats. Am J Physiol Endocrinol Metab 289: E328-336.

22. Golfman L, Dixon IM, Takeda N, Chapman D and Dhalla NS (1999) Differential changes in cardiac myofibrillar and sarcoplasmic reticular gene expression in alloxan-induced diabetes. Molecular and cellular biochemistry 200: 15-25.

23. Gower WR, Jr., San Miguel GI, Carter GM, Hassan I, Farese RV, et al. (2003) Atrial natriuretic hormone prohormone gene expression in cardiac and extra-cardiac tissues of diabetic Goto-Kakizaki rats. Molecular and cellular biochemistry 252: 263-271.

24. Gronholm T, Cheng ZJ, Palojoki E, Eriksson A, Backlund T, et al. (2005) Vasopeptidase inhibition has beneficial cardiac effects in spontaneously diabetic Goto-Kakizaki rats. European journal of pharmacology 519: 267-276.

25. Guglielmino K, Jackson K, Harris TR, Vu V, Dong H, et al. (2012) Pharmacological inhibition of soluble epoxide hydrolase provides cardioprotection in hyperglycemic rats. Am J Physiol Heart Circ Physiol 303: H853-862.

26. Gurusamy N, Watanabe K, Ma M, Zhang S, Muslin AJ, et al. (2005) Inactivation of 14-3-3 protein exacerbates cardiac hypertrophy and fibrosis through enhanced expression of protein kinase C beta 2 in experimental diabetes. Biological & pharmaceutical bulletin 28: 957-962.

27. Haddad F, Bodell PW, McCue SA and Baldwin KM (1997) Effects of diabetes on rodent cardiac thyroid hormone receptor and isomyosin expression. The American journal of physiology 272: E856-863.

28. Haddad F, Masatsugu M, Bodell PW, Qin A, McCue SA, et al. (1997) Role of thyroid hormone and insulin in control of cardiac isomyosin expression. Journal of molecular and cellular cardiology 29: 559-569.

29. Hofmann PA, Menon V and Gannaway KF (1995) Effects of diabetes on isometric tension as a function of [Ca2+] and pH in rat skinned cardiac myocytes. The American journal of physiology 269: H1656-1663.

30. Howarth FC, Qureshi MA, Hassan Z, Al Kury LT, Isaev D, et al. (2011) Changing pattern of gene expression is associated with ventricular myocyte dysfunction and altered mechanisms of Ca2+ signalling in young type 2 Zucker diabetic fatty rat heart. Experimental physiology 96: 325-337.

31. Howarth FC, Adem A, Adeghate EA, Al Ali NA, Al Bastaki AM, et al. (2005) Distribution of atrial natriuretic peptide and its effects on contraction and intracellular calcium in ventricular myocytes from streptozotocin-induced diabetic rat. Peptides 26: 691-700.

32. Jin P, Zhang X, Wu Y, Li L, Yin Q, et al. (2010) Streptozotocin-induced diabetic rat-derived bone marrow mesenchymal stem cells have impaired abilities in proliferation, paracrine, antiapoptosis, and myogenic differentiation. Transplantation proceedings 42: 2745-2752.

33. Kain V, Kumar S and Sitasawad SL (2011) Azelnidipine prevents cardiac dysfunction in streptozotocin-diabetic rats by reducing intracellular calcium accumulation, oxidative stress and apoptosis. Cardiovascular diabetology 10: 97.

34. Kaminski KA, Szepietowska B, Bonda T, Kozuch M, Mencel J, et al. (2009) CCN2 protein is an announcing marker for cardiac remodeling following STZ-induced moderate hyperglycemia in mice. Pharmacological reports : PR 61: 496-503.

35. Koka S, Xi L and Kukreja RC (2012) Chronic treatment with long acting phosphodiesterase-5 inhibitor tadalafil alters proteomic changes associated with cytoskeletal rearrangement and redox regulation in Type 2 diabetic hearts. Basic research in cardiology 107: 249.

36. Kralik PM, Ye G, Metreveli NS, Shem X and Epstein PN (2005) Cardiomyocyte dysfunction in models of type 1 and type 2 diabetes. Cardiovascular toxicology 5: 285-292.

37. Kruger M, Babicz K, von Frieling-Salewsky M and Linke WA (2010) Insulin signaling regulates cardiac titin properties in heart development and diabetic cardiomyopathy. Journal of molecular and cellular cardiology 48: 910-916.

38. Ku PM, Chen LJ, Liang JR, Cheng KC, Li YX, et al. (2011) Molecular role of GATA binding protein 4 (GATA-4) in hyperglycemia-induced reduction of cardiac contractility. Cardiovascular diabetology 10: 57.

39. LaRocca TJ, Fabris F, Chen J, Benhayon D, Zhang S, et al. (2012) Na+/Ca2+ exchanger-1 protects against systolic failure in the Akitains2 model of diabetic cardiomyopathy via a CXCR4/NF-kappaB pathway. Am J Physiol Heart Circ Physiol 303: H353-367.

40. Le Douairon Lahaye S, Gratas-Delamarche A, Malarde L, Zguira S, Vincent S, et al. (2012) Combined insulin treatment and intense exercise training improved basal cardiac function and Ca(2+)-cycling proteins expression in type 1 diabetic rats. Applied physiology, nutrition, and metabolism = Physiologie appliquee, nutrition et metabolisme 37: 53-62.

41. Li Q, Hueckstaedt LK and Ren J (2009) The protease inhibitor UCF-101 ameliorates streptozotocin-induced mouse cardiomyocyte contractile dysfunction in vitro: role of AMP-activated protein kinase. Experimental physiology 94: 984-994.

42. Ligeti L, Szenczi O, Prestia CM, Szabo C, Horvath K, et al. (2006) Altered calcium handling is an early sign of streptozotocin-induced diabetic cardiomyopathy. International journal of molecular medicine 17: 1035-1043.

43. Louhelainen M, Vahtola E, Forsten H, Merasto S, Kyto V, et al. (2009) Oral levosimendan prevents postinfarct heart failure and cardiac remodeling in diabetic Goto-Kakizaki rats. Journal of hypertension 27: 2094-2107.

44. Maalouf RM, Eid AA, Gorin YC, Block K, Escobar GP, et al. (2012) Nox4-derived reactive oxygen species mediate cardiomyocyte injury in early type 1 diabetes. American journal of physiology Cell physiology 302: C597-604.

45. Marsh SA, Dell'Italia LJ and Chatham JC (2011) Activation of the hexosamine biosynthesis pathway and protein O-GlcNAcylation modulate hypertrophic and cell signaling pathways in cardiomyocytes from diabetic mice. Amino Acids 40: 819-828.

46. Matsubara H, Mori Y, Yamamoto J and Inada M (1990) Diabetes-induced alterations in atrial natriuretic peptide gene expression in Wistar-Kyoto and spontaneously hypertensive rats. Circ Res 67: 803-813.

47. Mifune H, Suzuki S, Honda J, Kobayashi Y, Noda Y, et al. (1992) Atrial natriuretic peptide (ANP): a study of ANP and its mRNA in cardiocytes, and of plasma ANP levels in non-obese diabetic mice. Cell and tissue research 267: 267-272.

48. Miklos Z, Kemecsei P, Biro T, Marincsak R, Toth BI, et al. (2012) Early cardiac dysfunction is rescued by upregulation of SERCA2a pump activity in a rat model of metabolic syndrome. Acta physiologica 205: 381-393.

49. Morris GS, Prevost MC and Nelson AG (1996) Morderate diabetes alters myosin isoenzyme distribution in cardiac but not skeletal muscle of male rats. Life Sci 58: 833-838.

50. Nielsen LB, Bartels ED and Bollano E (2002) Overexpression of apolipoprotein B in the heart impedes cardiac triglyceride accumulation and development of cardiac dysfunction in diabetic mice. The Journal of biological chemistry 277: 27014-27020.

51. Ou HC, Tzang BS, Chang MH, Liu CT, Liu HW, et al. (2010) Cardiac contractile dysfunction and apoptosis in streptozotocin-induced diabetic rats are ameliorated by garlic oil supplementation. Journal of agricultural and food chemistry 58: 10347-10355.

52. Paulson DJ, Gupta M, Zak R and Zhao J (1992) Effects of exercise training and diabetes on cardiac myosin heavy chain composition. Molecular and cellular biochemistry 117: 175-179.

53. Pellegrino MA, Patrini C, Pasini E, Brocca L, Flati V, et al. (2008) Amino acid supplementation counteracts metabolic and functional damage in the diabetic rat heart. The American journal of cardiology 101: 49E-56E.

54. Pelzer T, Jazbutyte V, Arias-Loza PA, Segerer S, Lichtenwald M, et al. (2005) Pioglitazone reverses down-regulation of cardiac PPARgamma expression in Zucker diabetic fatty rats. Biochem Biophys Res Commun 329: 726-732.

55. Qi MY, Xia HJ, Dai DZ and Dai Y (2006) A novel endothelin receptor antagonist CPU0213 improves diabetic cardiac insufficiency attributed to up-regulation of the expression of FKBP12.6, SERCA2a, and PLB in rats. J Cardiovasc Pharmacol 47: 729-735.

56. Rajesh M, Batkai S, Kechrid M, Mukhopadhyay P, Lee WS, et al. (2012) Cannabinoid 1 receptor promotes cardiac dysfunction, oxidative stress, inflammation, and fibrosis in diabetic cardiomyopathy. Diabetes 61: 716-727.

57. Ritchie RH, Love JE, Huynh K, Bernardo BC, Henstridge DC, et al. (2012) Enhanced phosphoinositide 3-kinase(p110alpha) activity prevents diabetes-induced cardiomyopathy and superoxide generation in a mouse model of diabetes. Diabetologia 55: 3369-3381.

58. Rosenkranz AC, Hood SG, Woods RL, Dusting GJ and Ritchie RH (2003) B-type natriuretic peptide prevents acute hypertrophic responses in the diabetic rat heart: importance of cyclic GMP. Diabetes 52: 2389-2395.

59. Ruzicska E, Foldes G, Lako-Futo Z, Sarman B, Wellmann J, et al. (2004) Cardiac gene expression of natriuretic substances is altered in streptozotocin-induced diabetes during angiotensin II-induced pressure overload. Journal of hypertension 22: 1191-1200.

60. Sakata S, Lebeche D, Sakata Y, Sakata N, Chemaly ER, et al. (2007) Transcoronary gene transfer of SERCA2a increases coronary blood flow and decreases cardiomyocyte size in a type 2 diabetic rat model. Am J Physiol Heart Circ Physiol 292: H1204-1207.

61. Sakata S, Lebeche D, Sakata Y, Sakata N, Chemaly ER, et al. (2006) Mechanical and metabolic rescue in a type II diabetes model of cardiomyopathy by targeted gene transfer. Molecular therapy : the journal of the American Society of Gene Therapy 13: 987-996.

62. Sen S, Chen S, Wu Y, Feng B, Lui EK, et al. (2013) Preventive effects of North American ginseng (Panax quinquefolius) on diabetic retinopathy and cardiomyopathy. Phytotherapy research : PTR 27: 290-298.

63. Shah A, Oh YB, Shan G, Song CH, Park BH, et al. (2010) Angiotensin-(1-7) attenuates hyposmolarity-induced ANP secretion via the Na+-K+ pump. Peptides 31: 1779-1785.

64. Shao CH, Capek HL, Patel KP, Wang M, Tang K, et al. (2011) Carbonylation contributes to SERCA2a activity loss and diastolic dysfunction in a rat model of type 1 diabetes. Diabetes 60: 947-959.

65. Sharma V, Dhillon P, Wambolt R, Parsons H, Brownsey R, et al. (2008) Metoprolol improves cardiac function and modulates cardiac metabolism in the streptozotocin-diabetic rat. Am J Physiol Heart Circ Physiol 294: H1609-1620.

66. Sheikh AQ, Hurley JR, Huang W, Taghian T, Kogan A, et al. (2012) Diabetes alters intracellular calcium transients in cardiac endothelial cells. PloS one 7: e36840.

67. Soetikno V, Sari FR, Sukumaran V, Lakshmanan AP, Mito S, et al. (2012) Curcumin prevents diabetic cardiomyopathy in streptozotocin-induced diabetic rats: possible involvement of PKC-MAPK signaling pathway. European journal of pharmaceutical sciences : official journal of the European Federation for Pharmaceutical Sciences 47: 604-614.

68. Soman S, Manju CS, Rauf AA, Indira M and Rajamanickam C (2011) Role of cardiac isoform of alpha-2 macroglobulin in diabetic myocardium. Molecular and cellular biochemistry 350: 229-235.

69. Sulaiman M, Matta MJ, Sunderesan NR, Gupta MP, Periasamy M, et al. (2010) Resveratrol, an activator of SIRT1, upregulates sarcoplasmic calcium ATPase and improves cardiac function in diabetic cardiomyopathy. Am J Physiol Heart Circ Physiol 298: H833-843.

70. Talior-Volodarsky I, Connelly KA, Arora PD, Gullberg D and McCulloch CA (2012) alpha11 integrin stimulates myofibroblast differentiation in diabetic cardiomyopathy. Cardiovasc Res 96: 265-275.

71. Thandavarayan RA, Giridharan VV, Sari FR, Arumugam S, Veeraveedu PT, et al. (2011) Depletion of 14-3-3 protein exacerbates cardiac oxidative stress, inflammation and remodeling process via modulation of MAPK/NF-kB signaling pathways after streptozotocin-induced diabetes mellitus. Cellular physiology and biochemistry : international journal of experimental cellular physiology, biochemistry, and pharmacology 28: 911-922.

72. Thawornkaiwong A, Pantharanontaga J and Wattanapermpool J (2007) Hypersensitivity of myofilament response to Ca2+ in association with maladaptation of estrogen-deficient heart under diabetes complication. American journal of physiology Regulatory, integrative and comparative physiology 292: R844-851.

73. Tschope C, Spillmann F, Rehfeld U, Koch M, Westermann D, et al. (2004) Improvement of defective sarcoplasmic reticulum Ca2+ transport in diabetic heart of transgenic rats expressing the human kallikrein-1 gene. FASEB journal : official publication of the Federation of American Societies for Experimental Biology 18: 1967-1969.

74. Van Linthout S, Seeland U, Riad A, Eckhardt O, Hohl M, et al. (2008) Reduced MMP-2 activity contributes to cardiac fibrosis in experimental diabetic cardiomyopathy. Basic research in cardiology 103: 319-327.

75. Vasanji Z, Dhalla NS and Netticadan T (2004) Increased inhibition of SERCA2 by phospholamban in the type I diabetic heart. Molecular and cellular biochemistry 261: 245-249.

76. Vetter R, Rehfeld U, Reissfelder C, Weiss W, Wagner KD, et al. (2002) Transgenic overexpression of the sarcoplasmic reticulum Ca2+ATPase improves reticular Ca2+ handling in normal and diabetic rat hearts. FASEB journal : official publication of the Federation of American Societies for Experimental Biology 16: 1657-1659.

77. Wang M, Zhang WB, Zhu JH, Fu GS and Zhou BQ (2010) Breviscapine ameliorates cardiac dysfunction and regulates the myocardial Ca(2+)-cycling proteins in streptozotocin-induced diabetic rats. Acta diabetologica 47 Suppl 1: 209-218.

78. Wang YQ and Yao MH (2009) Effects of chromium picolinate on glucose uptake in insulin-resistant 3T3-L1 adipocytes involve activation of p38 MAPK. The Journal of nutritional biochemistry 20: 982-991.

79. Wang Y, Ebermann L, Sterner-Kock A, Wika S, Schultheiss HP, et al. (2009) Myocardial overexpression of adenine nucleotide translocase 1 ameliorates diabetic cardiomyopathy in mice. Experimental physiology 94: 220-227.

80. Wold LE, Dutta K, Mason MM, Ren J, Cala SE, et al. (2005) Impaired SERCA function contributes to cardiomyocyte dysfunction in insulin resistant rats. Journal of molecular and cellular cardiology 39: 297-307.

81. Wu SQ, Kwan CY and Tang F (1998) Streptozotocin-induced diabetes has differential effects on atrial natriuretic peptide synthesis in the rat atrium and ventricle: a study by solution-hybridization-RNase protection assay. Diabetologia 41: 660-665.

82. Yano S, Tanigawa K, Suzuki S, Kobayashi Y, Shimada T, et al. (1991) Effect of diabetes mellitus on levels of atrial natriuretic hormone in plasma and the right atrium in the non-obese diabetic mouse. Acta endocrinologica 124: 595-601.

83. Yeih DF, Yeh HI, Hsin HT, Lin LY, Chiang FT, et al. (2009) Dimethylthiourea normalizes velocity-dependent, but not force-dependent, index of ventricular performance in diabetic rats: role of myosin heavy chain isozyme. Am J Physiol Heart Circ Physiol 297: H1411-1420.

84. Yi T, Cheema Y, Tremble SM, Bell SP, Chen Z, et al. (2012) Zinc-induced cardiomyocyte relaxation in a rat model of hyperglycemia is independent of myosin isoform. Cardiovascular diabetology 11: 135.

85. Yokoe S, Asahi M, Takeda T, Otsu K, Taniguchi N, et al. (2010) Inhibition of phospholamban phosphorylation by O-GlcNAcylation: implications for diabetic cardiomyopathy. Glycobiology 20: 1217-1226.

86. Young ME, Wilson CR, Razeghi P, Guthrie PH and Taegtmeyer H (2002) Alterations of the circadian clock in the heart by streptozotocin-induced diabetes. Journal of molecular and cellular cardiology 34: 223-231.

87. Yue P, Arai T, Terashima M, Sheikh AY, Cao F, et al. (2007) Magnetic resonance imaging of progressive cardiomyopathic changes in the db/db mouse. Am J Physiol Heart Circ Physiol 292: H2106-2118.

88. Zarain-Herzberg A, Yano K, Elimban V and Dhalla NS (1994) Cardiac sarcoplasmic reticulum Ca(2+)-ATPase expression in streptozotocin-induced diabetic rat heart. Biochem Biophys Res Commun 203: 113-120.

89. Zhang J, Zhang BH, Yu YR, Tang CS and Qi YF (2011) Adrenomedullin protects against fructose-induced insulin resistance and myocardial hypertrophy in rats. Peptides 32: 1415-1421.

90. Zhong Y, Ahmed S, Grupp IL and Matlib MA (2001) Altered SR protein expression associated with contractile dysfunction in diabetic rat hearts. Am J Physiol Heart Circ Physiol 281: H1137-1147.

91. Zhou H, Li YJ, Wang M, Zhang LH, Guo BY, et al. (2011) Involvement of RhoA/ROCK in myocardial fibrosis in a rat model of type 2 diabetes. Acta pharmacologica Sinica 32: 999-1008.

92. Zu L, Bedja D, Fox-Talbot K, Gabrielson KL, Van Kaer L, et al. (2010) Evidence for a role of immunoproteasomes in regulating cardiac muscle mass in diabetic mice. Journal of molecular and cellular cardiology 49: 5-15.
